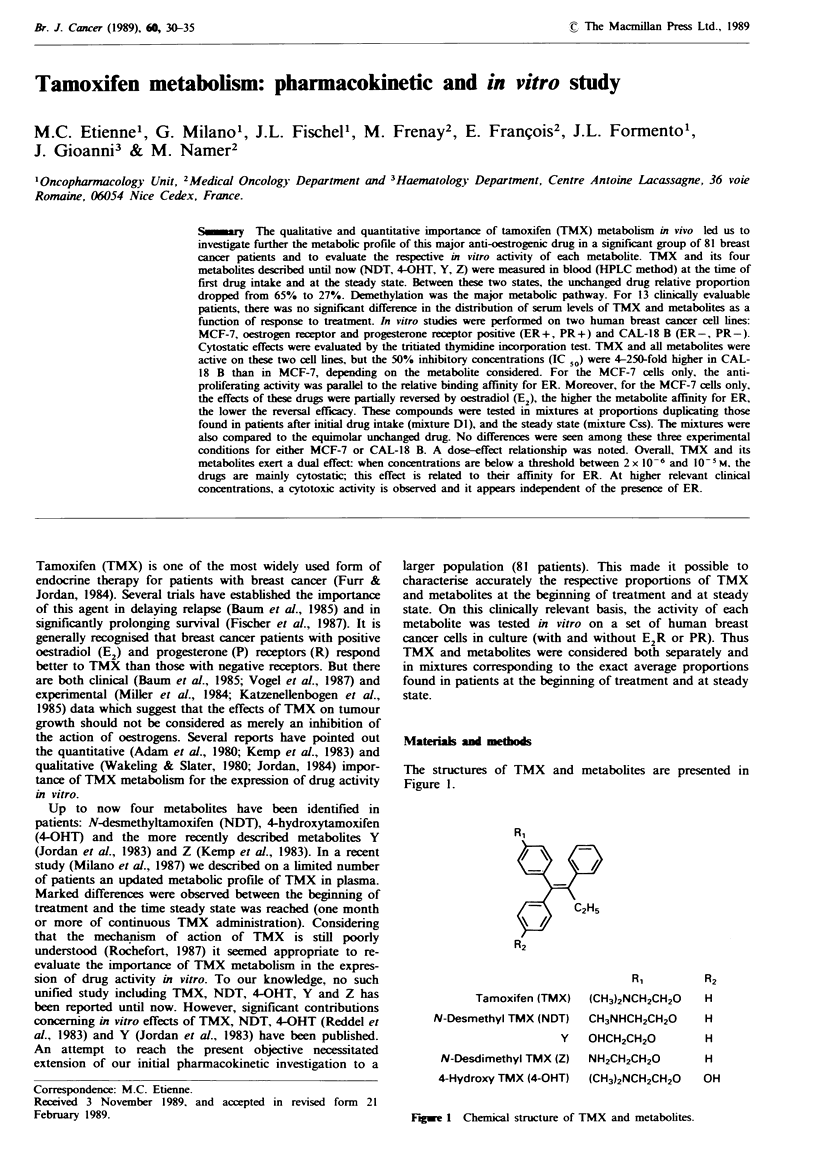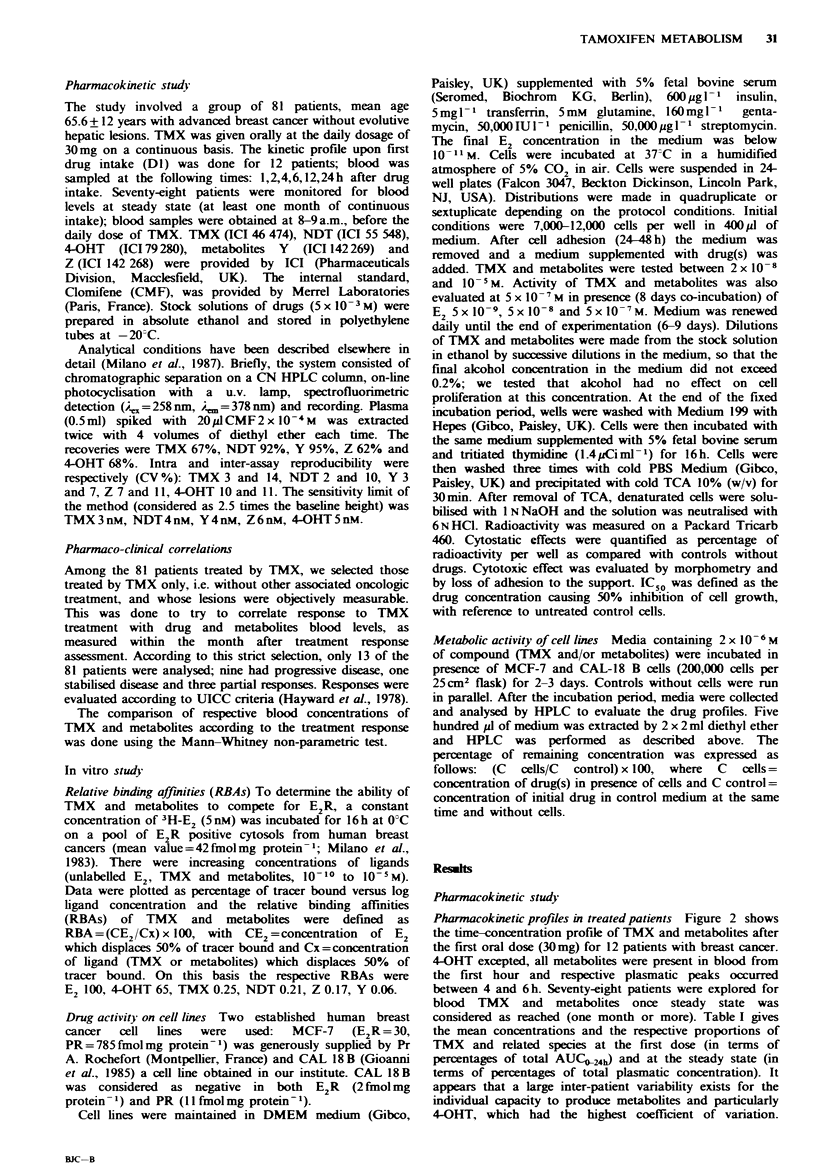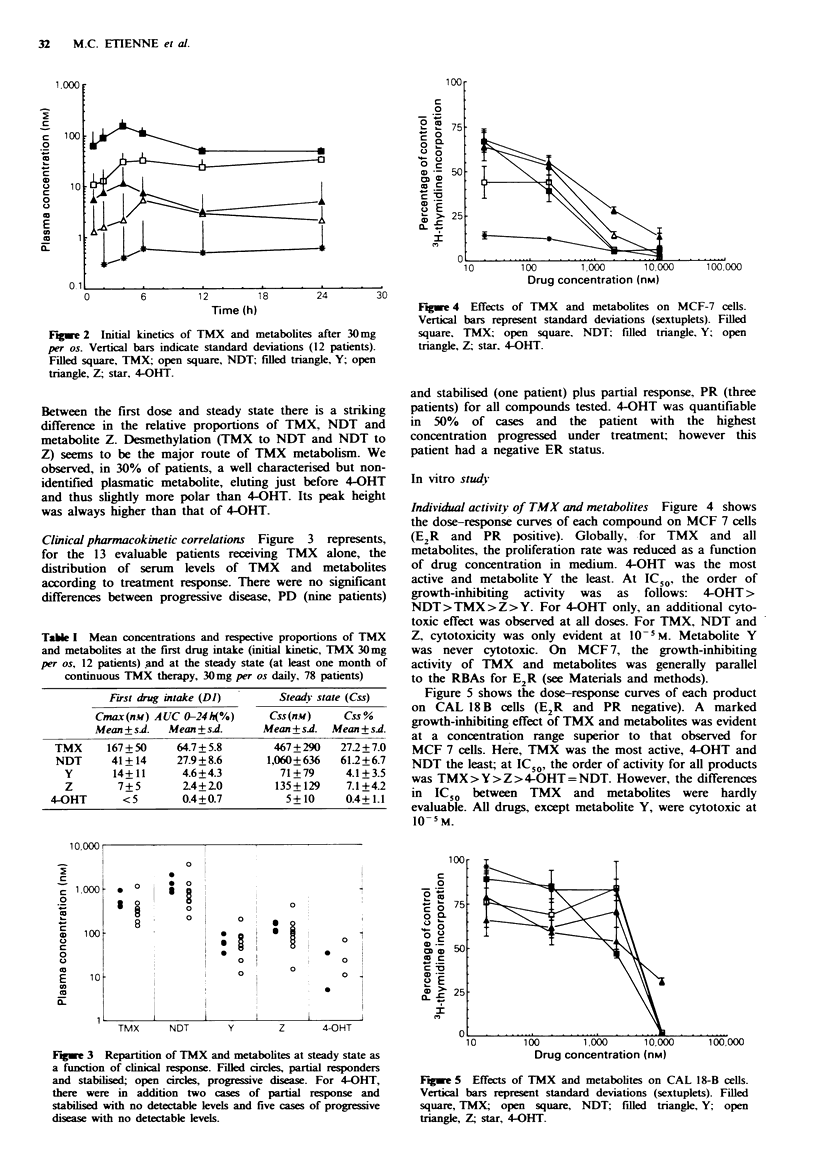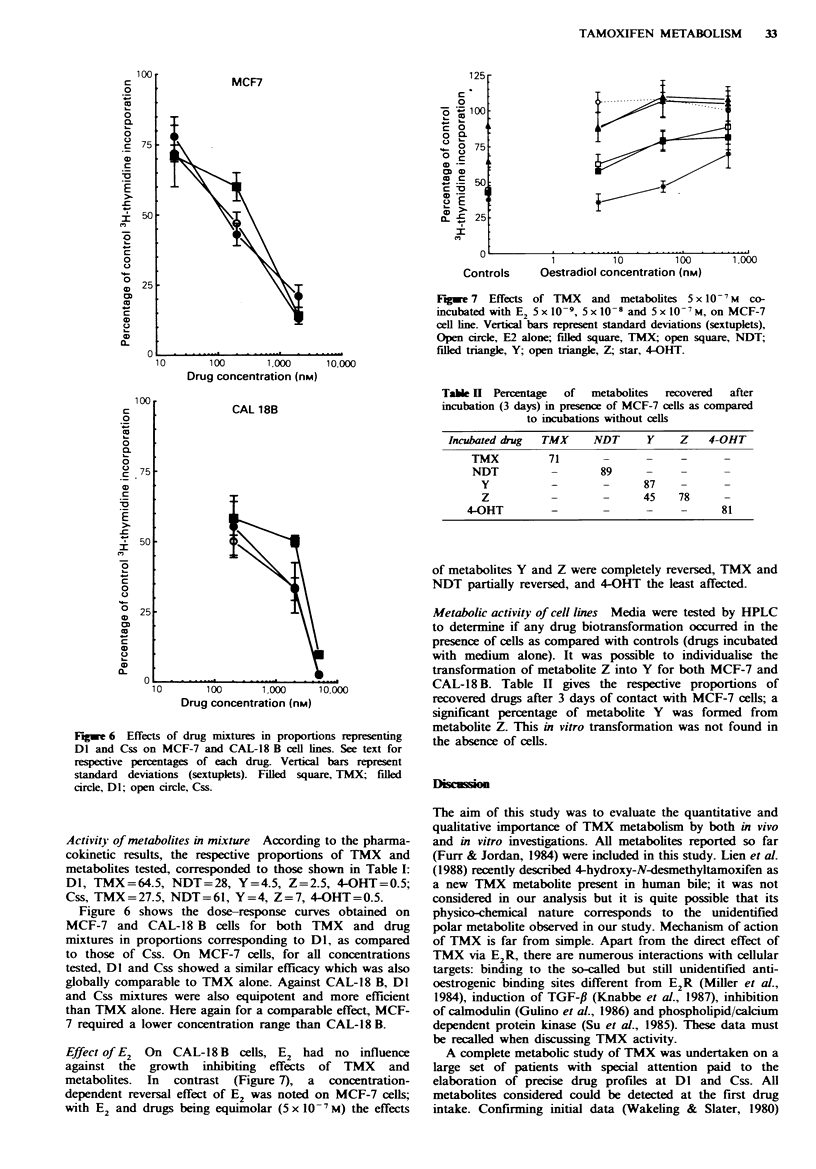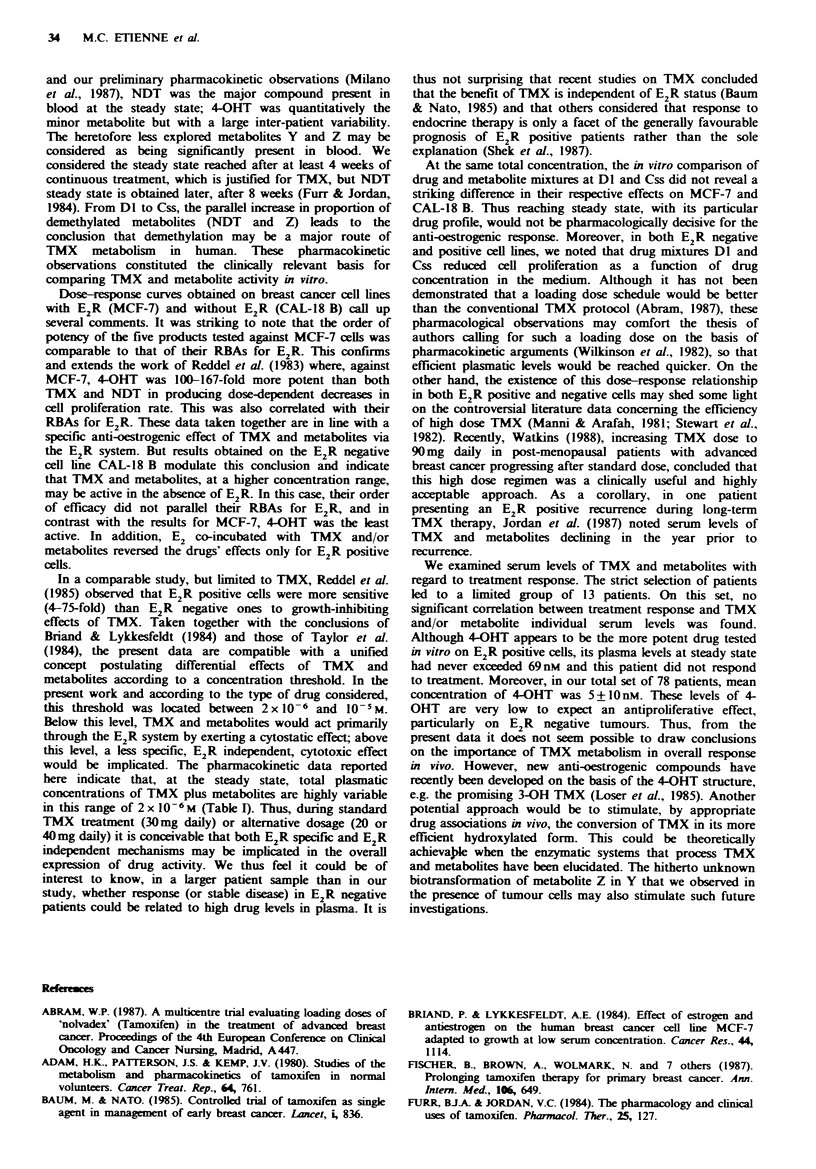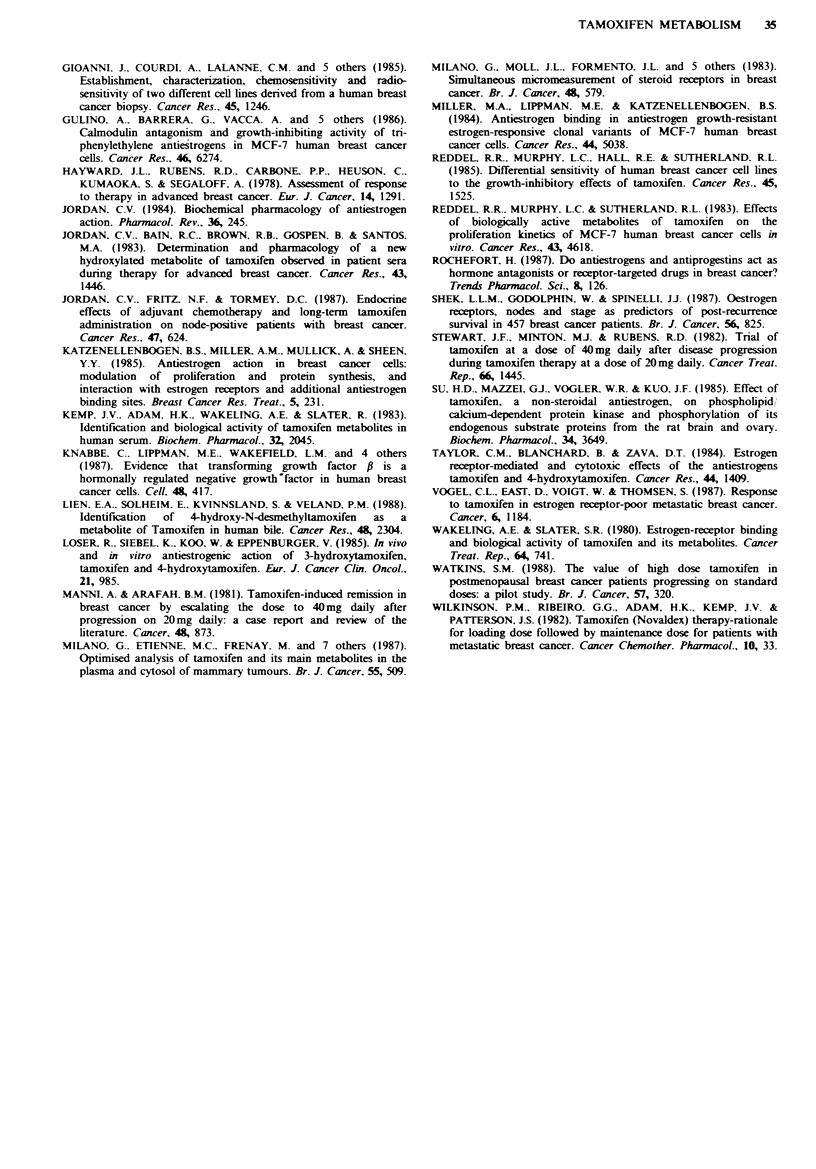# Tamoxifen metabolism: pharmacokinetic and in vitro study.

**DOI:** 10.1038/bjc.1989.214

**Published:** 1989-07

**Authors:** M. C. Etienne, G. Milano, J. L. Fischel, M. Frenay, E. FranÃ§ois, J. L. Formento, J. Gioanni, M. Namer

**Affiliations:** Oncopharmacology Unit, Centre Antoine Lacassagne, Nice, France.

## Abstract

The qualitative and quantitative importance of tamoxifen (TMX) metabolism in vivo led us to investigate further the metabolic profile of this major anti-oestrogenic drug in a significant group of 81 breast cancer patients and to evaluate the respective in vitro activity of each metabolite. TMX and its four metabolites described until now (NDT, 4-OHT, Y, Z) were measured in blood (HPLC method) at the time of first drug intake and at the steady state. Between these two states, the unchanged drug relative proportion dropped from 65% to 27%. Demethylation was the major metabolic pathway. For 13 clinically evaluable patients, there was no significant difference in the distribution of serum levels of TMX and metabolites as a function of response to treatment. In vitro studies were performed on two human breast cancer cell lines: MCF-7, oestrogen receptor and progesterone receptor positive (ER+, PR+) and CAL-18 B (ER-, PR-). Cytostatic effects were evaluated by the tritiated thymidine incorporation test. TMX and all metabolites were active on these two cell lines, but the 50% inhibitory concentrations (IC50) were 4-250-fold higher in CAL-18 B than in MCF-7, depending on the metabolite considered. For the MCF-7 cells only, the antiproliferating activity was parallel to the relative binding affinity for ER. Moreover, for the MCF-7 cells only, the effects of these drugs were partially reversed by oestradiol (E2), the higher the metabolite affinity for ER, the lower the reversal efficacy. These compounds were tested in mixtures at proportions duplicating those found in patients after initial drug intake (mixture D1), and the steady state (mixture Css). The mixtures were also compared to the equimolar unchanged drug. No differences were seen among these three experimental conditions for either MCF-7 or CAL-18 B. A dose-effect relationship was noted. Overall, TMX and its metabolites exert a dual effect: when concentrations are below a threshold between 2 x 10(-6) and 10(-5) M, the drugs are mainly cytostatic; this effect is related to their affinity for ER. At higher relevant clinical concentrations, a cytotoxic activity is observed and it appears independent of the presence of ER.


*Br. J. Cancer* (1989), **60**, 30–35
© The Macmillan Press Ltd., 1989

### Tamoxifen metabolism: pharmacokinetic and *in vitro* study

M.C. Etienne[1], G. Milano[1], J.L. Fischel[1], M. Frenay[2], E. François[2], J.L. Formento[1], J. Gioanni[3] & M. Namer[2]

[1]*Oncopharmacology Unit,* [2]*Medical Oncology Department and* [3]*Haematology Department, Centre Antoine Lacassagne, 36 voie Romaine, 06054 Nice Cedex, France.*

**Summary** The qualitative and quantitative importance of tamoxifen (TMX) metabolism *in vivo* led us to investigate further the metabolic profile of this major anti-oestrogenic drug in a significant group of 81 breast cancer patients and to evaluate the respective *in vitro* activity of each metabolite. TMX and its four metabolites described until now (NDT, 4-OHT, Y, Z) were measured in blood (HPLC method) at the time of first drug intake and at the steady state. Between these two states, the unchanged drug relative proportion dropped from 65% to 27%. Demethylation was the major metabolic pathway. For 13 clinically evaluable patients, there was no significant difference in the distribution of serum levels of TMX and metabolites as a function of response to treatment. *In vitro* studies were performed on two human breast cancer cell lines: MCF-7, oestrogen receptor and progesterone receptor positive (ER+, PR+) and CAL-18 B (ER−, PR−). Cytostatic effects were evaluated by the tritiated thymidine incorporation test. TMX and all metabolites were active on these two cell lines, but the 50% inhibitory concentrations ($IC_{50}$) were 4–250-fold higher in CAL-18 B than in MCF-7, depending on the metabolite considered. For the MCF-7 cells only, the anti-proliferating activity was parallel to the relative binding affinity for ER. Moreover, for the MCF-7 cells only, the effects of these drugs were partially reversed by oestradiol ($E_2$), the higher the metabolite affinity for ER, the lower the reversal efficacy. These compounds were tested in mixtures at proportions duplicating those found in patients after initial drug intake (mixture D1), and the steady state (mixture Css). The mixtures were also compared to the equimolar unchanged drug. No differences were seen among these three experimental conditions for either MCF-7 or CAL-18 B. A dose–effect relationship was noted. Overall, TMX and its metabolites exert a dual effect: when concentrations are below a threshold between $2 \times 10^{-6}$ and $10^{-5}$ M, the drugs are mainly cytostatic; this effect is related to their affinity for ER. At higher relevant clinical concentrations, a cytotoxic activity is observed and it appears independent of the presence of ER.

Tamoxifen (TMX) is one of the most widely used form of endocrine therapy for patients with breast cancer (Furr & Jordan, 1984). Several trials have established the importance of this agent in delaying relapse (Baum *et al.*, 1985) and in significantly prolonging survival (Fischer *et al.*, 1987). It is generally recognised that breast cancer patients with positive oestradiol ($E_2$) and progesterone (P) receptors (R) respond better to TMX than those with negative receptors. But there are both clinical (Baum *et al.*, 1985; Vogel *et al.*, 1987) and experimental (Miller *et al.*, 1984; Katzenellenbogen *et al.*, 1985) data which suggest that the effects of TMX on tumour growth should not be considered as merely an inhibition of the action of oestrogens. Several reports have pointed out the quantitative (Adam *et al.*, 1980; Kemp *et al.*, 1983) and qualitative (Wakeling & Slater, 1980; Jordan, 1984) importance of TMX metabolism for the expression of drug activity *in vitro*.

Up to now four metabolites have been identified in patients: *N*-desmethyltamoxifen (NDT), 4-hydroxytamoxifen (4-OHT) and the more recently described metabolites Y (Jordan *et al.*, 1983) and Z (Kemp *et al.*, 1983). In a recent study (Milano *et al.*, 1987) we described on a limited number of patients an updated metabolic profile of TMX in plasma. Marked differences were observed between the beginning of treatment and the time steady state was reached (one month or more of continuous TMX administration). Considering that the mechanism of action of TMX is still poorly understood (Rochefort, 1987) it seemed appropriate to re-evaluate the importance of TMX metabolism in the expression of drug activity *in vitro*. To our knowledge, no such unified study including TMX, NDT, 4-OHT, Y and Z has been reported until now. However, significant contributions concerning *in vitro* effects of TMX, NDT, 4-OHT (Reddel *et al.*, 1983) and Y (Jordan *et al.*, 1983) have been published. An attempt to reach the present objective necessitated extension of our initial pharmacokinetic investigation to a larger population (81 patients). This made it possible to characterise accurately the respective proportions of TMX and metabolites at the beginning of treatment and at steady state. On this clinically relevant basis, the activity of each metabolite was tested *in vitro* on a set of human breast cancer cells in culture (with and without $E_2R$ or PR). Thus TMX and metabolites were considered both separately and in mixtures corresponding to the exact average proportions found in patients at the beginning of treatment and at steady state.

Correspondence: M.C. Etienne.
Received 3 November 1989, and accepted in revised form 21 February 1989.

### Materials and methods

The structures of TMX and metabolites are presented in Figure 1.

| | $R_1$ | $R_2$ |
|---|---|---|
| Tamoxifen (TMX) | $(CH_3)_2NCH_2CH_2O$ | H |
| *N*-Desmethyl TMX (NDT) | $CH_3NHCH_2CH_2O$ | H |
| Y | $OHCH_2CH_2O$ | H |
| *N*-Desdimethyl TMX (Z) | $NH_2CH_2CH_2O$ | H |
| 4-Hydroxy TMX (4-OHT) | $(CH_3)_2NCH_2CH_2O$ | OH |

**Figure 1** Chemical structure of TMX and metabolites.

*Pharmacokinetic study*

The study involved a group of 81 patients, mean age 65.6 ± 12 years with advanced breast cancer without evolutive hepatic lesions. TMX was given orally at the daily dosage of 30 mg on a continuous basis. The kinetic profile upon first drug intake (D1) was done for 12 patients; blood was sampled at the following times: 1,2,4,6,12,24 h after drug intake. Seventy-eight patients were monitored for blood levels at steady state (at least one month of continuous intake); blood samples were obtained at 8–9 a.m., before the daily dose of TMX. TMX (ICI 46 474), NDT (ICI 55 548), 4-OHT (ICI 79 280), metabolites Y (ICI 142 269) and Z (ICI 142 268) were provided by ICI (Pharmaceuticals Division, Macclesfield, UK). The internal standard, Clomifene (CMF), was provided by Merrel Laboratories (Paris, France). Stock solutions of drugs ($5 \times 10^{-3}$ M) were prepared in absolute ethanol and stored in polyethylene tubes at −20°C.

Analytical conditions have been described elsewhere in detail (Milano *et al.*, 1987). Briefly, the system consisted of chromatographic separation on a CN HPLC column, on-line photocyclisation with a u.v. lamp, spectrofluorimetric detection ($\lambda_{ex} = 258$ nm, $\lambda_{em} = 378$ nm) and recording. Plasma (0.5 ml) spiked with 20 µl CMF $2 \times 10^{-4}$ M was extracted twice with 4 volumes of diethyl ether each time. The recoveries were TMX 67%, NDT 92%, Y 95%, Z 62% and 4-OHT 68%. Intra and inter-assay reproducibility were respectively (CV %): TMX 3 and 14, NDT 2 and 10, Y 3 and 7, Z 7 and 11, 4-OHT 10 and 11. The sensitivity limit of the method (considered as 2.5 times the baseline height) was TMX 3 nM, NDT 4 nM, Y 4 nM, Z 6 nM, 4-OHT 5 nM.

*Pharmaco-clinical correlations*

Among the 81 patients treated by TMX, we selected those treated by TMX only, i.e. without other associated oncologic treatment, and whose lesions were objectively measurable. This was done to try to correlate response to TMX treatment with drug and metabolites blood levels, as measured within the month after treatment response assessment. According to this strict selection, only 13 of the 81 patients were analysed; nine had progressive disease, one stabilised disease and three partial responses. Responses were evaluated according to UICC criteria (Hayward *et al.*, 1978).

The comparison of respective blood concentrations of TMX and metabolites according to the treatment response was done using the Mann–Whitney non-parametric test.

In vitro *study*

*Relative binding affinities (RBAs)* To determine the ability of TMX and metabolites to compete for $E_2R$, a constant concentration of $^3H$-$E_2$ (5 nM) was incubated for 16 h at 0°C on a pool of $E_2R$ positive cytosols from human breast cancers (mean value = 42 fmol mg protein$^{-1}$; Milano *et al.*, 1983). There were increasing concentrations of ligands (unlabelled $E_2$, TMX and metabolites, $10^{-10}$ to $10^{-5}$ M). Data were plotted as percentage of tracer bound versus log ligand concentration and the relative binding affinities (RBAs) of TMX and metabolites were defined as $RBA = (CE_2/Cx) \times 100$, with $CE_2$ = concentration of $E_2$ which displaces 50% of tracer bound and Cx = concentration of ligand (TMX or metabolites) which displaces 50% of tracer bound. On this basis the respective RBAs were $E_2$ 100, 4-OHT 65, TMX 0.25, NDT 0.21, Z 0.17, Y 0.06.

*Drug activity on cell lines* Two established human breast cancer cell lines were used: MCF-7 ($E_2R = 30$, PR = 785 fmol mg protein$^{-1}$) was generously supplied by Pr A. Rochefort (Montpellier, France) and CAL 18 B (Gioanni *et al.*, 1985) a cell line obtained in our institute. CAL 18 B was considered as negative in both $E_2R$ (2 fmol mg protein$^{-1}$) and PR (11 fmol mg protein$^{-1}$).

Cell lines were maintained in DMEM medium (Gibco, Paisley, UK) supplemented with 5% fetal bovine serum (Seromed, Biochrom KG, Berlin), 600 µg l$^{-1}$ insulin, 5 mg l$^{-1}$ transferrin, 5 mM glutamine, 160 mg l$^{-1}$ gentamycin, 50,000 IU l$^{-1}$ penicillin, 50,000 µg l$^{-1}$ streptomycin. The final $E_2$ concentration in the medium was below $10^{-11}$ M. Cells were incubated at 37°C in a humidified atmosphere of 5% $CO_2$ in air. Cells were suspended in 24-well plates (Falcon 3047, Beckton Dickinson, Lincoln Park, NJ, USA). Distributions were made in quadruplicate or sextuplicate depending on the protocol conditions. Initial conditions were 7,000–12,000 cells per well in 400 µl of medium. After cell adhesion (24–48 h) the medium was removed and a medium supplemented with drug(s) was added. TMX and metabolites were tested between $2 \times 10^{-8}$ and $10^{-5}$ M. Activity of TMX and metabolites was also evaluated at $5 \times 10^{-7}$ M in presence (8 days co-incubation) of $E_2$ $5 \times 10^{-9}$, $5 \times 10^{-8}$ and $5 \times 10^{-7}$ M. Medium was renewed daily until the end of experimentation (6–9 days). Dilutions of TMX and metabolites were made from the stock solution in ethanol by successive dilutions in the medium, so that the final alcohol concentration in the medium did not exceed 0.2%; we tested that alcohol had no effect on cell proliferation at this concentration. At the end of the fixed incubation period, wells were washed with Medium 199 with Hepes (Gibco, Paisley, UK). Cells were then incubated with the same medium supplemented with 5% fetal bovine serum and tritiated thymidine (1.4 µCi ml$^{-1}$) for 16 h. Cells were then washed three times with cold PBS Medium (Gibco, Paisley, UK) and precipitated with cold TCA 10% (w/v) for 30 min. After removal of TCA, denaturated cells were solubilised with 1 N NaOH and the solution was neutralised with 6 N HCl. Radioactivity was measured on a Packard Tricarb 460. Cytostatic effects were quantified as percentage of radioactivity per well as compared with controls without drugs. Cytotoxic effect was evaluated by morphometry and by loss of adhesion to the support. $IC_{50}$ was defined as the drug concentration causing 50% inhibition of cell growth, with reference to untreated control cells.

*Metabolic activity of cell lines* Media containing $2 \times 10^{-6}$ M of compound (TMX and/or metabolites) were incubated in presence of MCF-7 and CAL-18 B cells (200,000 cells per 25 cm$^2$ flask) for 2–3 days. Controls without cells were run in parallel. After the incubation period, media were collected and analysed by HPLC to evaluate the drug profiles. Five hundred µl of medium was extracted by 2 × 2 ml diethyl ether and HPLC was performed as described above. The percentage of remaining concentration was expressed as follows: (C cells/C control) × 100, where C cells = concentration of drug(s) in presence of cells and C control = concentration of initial drug in control medium at the same time and without cells.

### Results

*Pharmacokinetic study*

*Pharmacokinetic profiles in treated patients* Figure 2 shows the time–concentration profile of TMX and metabolites after the first oral dose (30 mg) for 12 patients with breast cancer. 4-OHT excepted, all metabolites were present in blood from the first hour and respective plasmatic peaks occurred between 4 and 6 h. Seventy-eight patients were explored for blood TMX and metabolites once steady state was considered as reached (one month or more). Table I gives the mean concentrations and the respective proportions of TMX and related species at the first dose (in terms of percentages of total $AUC_{0-24h}$) and at the steady state (in terms of percentages of total plasmatic concentration). It appears that a large inter-patient variability exists for the individual capacity to produce metabolites and particularly 4-OHT, which had the highest coefficient of variation.

Figure 2 Initial kinetics of TMX and metabolites after 30 mg *per os*. Vertical bars indicate standard deviations (12 patients). Filled square, TMX; open square, NDT; filled triangle, Y; open triangle, Z; star, 4-OHT.

Between the first dose and steady state there is a striking difference in the relative proportions of TMX, NDT and metabolite Z. Desmethylation (TMX to NDT and NDT to Z) seems to be the major route of TMX metabolism. We observed, in 30% of patients, a well characterised but non-identified plasmatic metabolite, eluting just before 4-OHT and thus slightly more polar than 4-OHT. Its peak height was always higher than that of 4-OHT.

*Clinical pharmacokinetic correlations* Figure 3 represents, for the 13 evaluable patients receiving TMX alone, the distribution of serum levels of TMX and metabolites according to treatment response. There were no significant differences between progressive disease, PD (nine patients)

Table I Mean concentrations and respective proportions of TMX and metabolites at the first drug intake (initial kinetic, TMX 30 mg *per os*, 12 patients) and at the steady state (at least one month of continuous TMX therapy, 30 mg *per os* daily, 78 patients)

| | *First drug intake (D1)* | | *Steady state (Css)* | |
|---|---|---|---|---|
| | *Cmax (nM)* *Mean ± s.d.* | *AUC 0–24 h(%)* *Mean ± s.d.* | *Css (nM)* *Mean ± s.d.* | *Css %* *Mean ± s.d.* |
| TMX | 167 ± 50 | 64.7 ± 5.8 | 467 ± 290 | 27.2 ± 7.0 |
| NDT | 41 ± 14 | 27.9 ± 8.6 | 1,060 ± 636 | 61.2 ± 6.7 |
| Y | 14 ± 11 | 4.6 ± 4.3 | 71 ± 79 | 4.1 ± 3.5 |
| Z | 7 ± 5 | 2.4 ± 2.0 | 135 ± 129 | 7.1 ± 4.2 |
| 4-OHT | < 5 | 0.4 ± 0.7 | 5 ± 10 | 0.4 ± 1.1 |

Figure 3 Repartition of TMX and metabolites at steady state as a function of clinical response. Filled circles, partial responders and stabilised; open circles, progressive disease. For 4-OHT, there were in addition two cases of partial response and stabilised with no detectable levels and five cases of progressive disease with no detectable levels.

Figure 4 Effects of TMX and metabolites on MCF-7 cells. Vertical bars represent standard deviations (sextuplets). Filled square, TMX; open square, NDT; filled triangle, Y; open triangle, Z; star, 4-OHT.

and stabilised (one patient) plus partial response, PR (three patients) for all compounds tested. 4-OHT was quantifiable in 50% of cases and the patient with the highest concentration progressed under treatment; however this patient had a negative ER status.

### In vitro *study*

*Individual activity of TMX and metabolites* Figure 4 shows the dose–response curves of each compound on MCF 7 cells ($E_2R$ and PR positive). Globally, for TMX and all metabolites, the proliferation rate was reduced as a function of drug concentration in medium. 4-OHT was the most active and metabolite Y the least. At $IC_{50}$, the order of growth-inhibiting activity was as follows: 4-OHT > NDT > TMX > Z > Y. For 4-OHT only, an additional cytotoxic effect was observed at all doses. For TMX, NDT and Z, cytotoxicity was only evident at $10^{-5}$ M. Metabolite Y was never cytotoxic. On MCF 7, the growth-inhibiting activity of TMX and metabolites was generally parallel to the RBAs for $E_2R$ (see Materials and methods).

Figure 5 shows the dose–response curves of each product on CAL 18 B cells ($E_2R$ and PR negative). A marked growth-inhibiting effect of TMX and metabolites was evident at a concentration range superior to that observed for MCF 7 cells. Here, TMX was the most active, 4-OHT and NDT the least; at $IC_{50}$, the order of activity for all products was TMX > Y > Z > 4-OHT = NDT. However, the differences in $IC_{50}$ between TMX and metabolites were hardly evaluable. All drugs, except metabolite Y, were cytotoxic at $10^{-5}$ M.

Figure 5 Effects of TMX and metabolites on CAL 18-B cells. Vertical bars represent standard deviations (sextuplets). Filled square, TMX; open square, NDT; filled triangle, Y; open triangle, Z; star, 4-OHT.

**Figure 6** Effects of drug mixtures in proportions representing D1 and Css on MCF-7 and CAL-18 B cell lines. See text for respective percentages of each drug. Vertical bars represent standard deviations (sextuplets). Filled square, TMX; filled circle, D1; open circle, Css.

**Figure 7** Effects of TMX and metabolites $5 \times 10^{-7}$ M co-incubated with $E_2$ $5 \times 10^{-9}$, $5 \times 10^{-8}$ and $5 \times 10^{-7}$ M, on MCF-7 cell line. Vertical bars represent standard deviations (sextuplets), Open circle, E2 alone; filled square, TMX; open square, NDT; filled triangle, Y; open triangle, Z; star, 4-OHT.

**Table II** Percentage of metabolites recovered after incubation (3 days) in presence of MCF-7 cells as compared to incubations without cells

| *Incubated drug* | *TMX* | *NDT* | *Y* | *Z* | *4-OHT* |
|---|---|---|---|---|---|
| TMX | 71 | – | – | – | – |
| NDT | – | 89 | – | – | – |
| Y | – | – | 87 | – | – |
| Z | – | – | 45 | 78 | – |
| 4-OHT | – | – | – | – | 81 |

*Activity of metabolites in mixture* According to the pharmacokinetic results, the respective proportions of TMX and metabolites tested, corresponded to those shown in Table I: D1, TMX=64.5, NDT=28, Y=4.5, Z=2.5, 4-OHT=0.5; Css, TMX=27.5, NDT=61, Y=4, Z=7, 4-OHT=0.5.

Figure 6 shows the dose–response curves obtained on MCF-7 and CAL-18 B cells for both TMX and drug mixtures in proportions corresponding to D1, as compared to those of Css. On MCF-7 cells, for all concentrations tested, D1 and Css showed a similar efficacy which was also globally comparable to TMX alone. Against CAL-18 B, D1 and Css mixtures were also equipotent and more efficient than TMX alone. Here again for a comparable effect, MCF-7 required a lower concentration range than CAL-18 B.

*Effect of $E_2$* On CAL-18 B cells, $E_2$ had no influence against the growth inhibiting effects of TMX and metabolites. In contrast (Figure 7), a concentration-dependent reversal effect of $E_2$ was noted on MCF-7 cells; with $E_2$ and drugs being equimolar ($5 \times 10^{-7}$ M) the effects of metabolites Y and Z were completely reversed, TMX and NDT partially reversed, and 4-OHT the least affected.

*Metabolic activity of cell lines* Media were tested by HPLC to determine if any drug biotransformation occurred in the presence of cells as compared with controls (drugs incubated with medium alone). It was possible to individualise the transformation of metabolite Z into Y for both MCF-7 and CAL-18 B. Table II gives the respective proportions of recovered drugs after 3 days of contact with MCF-7 cells; a significant percentage of metabolite Y was formed from metabolite Z. This *in vitro* transformation was not found in the absence of cells.

### Discussion

The aim of this study was to evaluate the quantitative and qualitative importance of TMX metabolism by both *in vivo* and *in vitro* investigations. All metabolites reported so far (Furr & Jordan, 1984) were included in this study. Lien *et al.* (1988) recently described 4-hydroxy-*N*-desmethyltamoxifen as a new TMX metabolite present in human bile; it was not considered in our analysis but it is quite possible that its physico-chemical nature corresponds to the unidentified polar metabolite observed in our study. Mechanism of action of TMX is far from simple. Apart from the direct effect of TMX via $E_2$R, there are numerous interactions with cellular targets: binding to the so-called but still unidentified anti-oestrogenic binding sites different from $E_2$R (Miller *et al.*, 1984), induction of TGF-$\beta$ (Knabbe *et al.*, 1987), inhibition of calmodulin (Gulino *et al.*, 1986) and phospholipid/calcium dependent protein kinase (Su *et al.*, 1985). These data must be recalled when discussing TMX activity.

A complete metabolic study of TMX was undertaken on a large set of patients with special attention paid to the elaboration of precise drug profiles at D1 and Css. All metabolites considered could be detected at the first drug intake. Confirming initial data (Wakeling & Slater, 1980)

and our preliminary pharmacokinetic observations (Milano *et al.*, 1987), NDT was the major compound present in blood at the steady state; 4-OHT was quantitatively the minor metabolite but with a large inter-patient variability. The heretofore less explored metabolites Y and Z may be considered as being significantly present in blood. We considered the steady state reached after at least 4 weeks of continuous treatment, which is justified for TMX, but NDT steady state is obtained later, after 8 weeks (Furr & Jordan, 1984). From D1 to Css, the parallel increase in proportion of demethylated metabolites (NDT and Z) leads to the conclusion that demethylation may be a major route of TMX metabolism in human. These pharmacokinetic observations constituted the clinically relevant basis for comparing TMX and metabolite activity *in vitro*.

Dose–response curves obtained on breast cancer cell lines with $E_2R$ (MCF-7) and without $E_2R$ (CAL-18 B) call up several comments. It was striking to note that the order of potency of the five products tested against MCF-7 cells was comparable to that of their RBAs for $E_2R$. This confirms and extends the work of Reddel *et al.* (1983) where, against MCF-7, 4-OHT was 100–167-fold more potent than both TMX and NDT in producing dose-dependent decreases in cell proliferation rate. This was also correlated with their RBAs for $E_2R$. These data taken together are in line with a specific anti-oestrogenic effect of TMX and metabolites via the $E_2R$ system. But results obtained on the $E_2R$ negative cell line CAL-18 B modulate this conclusion and indicate that TMX and metabolites, at a higher concentration range, may be active in the absence of $E_2R$. In this case, their order of efficacy did not parallel their RBAs for $E_2R$, and in contrast with the results for MCF-7, 4-OHT was the least active. In addition, $E_2$ co-incubated with TMX and/or metabolites reversed the drugs' effects only for $E_2R$ positive cells.

In a comparable study, but limited to TMX, Reddel *et al.* (1985) observed that $E_2R$ positive cells were more sensitive (4–75-fold) than $E_2R$ negative ones to growth-inhibiting effects of TMX. Taken together with the conclusions of Briand & Lykkesfeldt (1984) and those of Taylor *et al.* (1984), the present data are compatible with a unified concept postulating differential effects of TMX and metabolites according to a concentration threshold. In the present work and according to the type of drug considered, this threshold was located between $2\times10^{-6}$ and $10^{-5}$ M. Below this level, TMX and metabolites would act primarily through the $E_2R$ system by exerting a cytostatic effect; above this level, a less specific, $E_2R$ independent, cytotoxic effect would be implicated. The pharmacokinetic data reported here indicate that, at the steady state, total plasmatic concentrations of TMX plus metabolites are highly variable in this range of $2\times10^{-6}$ M (Table I). Thus, during standard TMX treatment (30 mg daily) or alternative dosage (20 or 40 mg daily) it is conceivable that both $E_2R$ specific and $E_2R$ independent mechanisms may be implicated in the overall expression of drug activity. We thus feel it could be of interest to know, in a larger patient sample than in our study, whether response (or stable disease) in $E_2R$ negative patients could be related to high drug levels in plasma. It is thus not surprising that recent studies on TMX concluded that the benefit of TMX is independent of $E_2R$ status (Baum & Nato, 1985) and that others considered that response to endocrine therapy is only a facet of the generally favourable prognosis of $E_2R$ positive patients rather than the sole explanation (Shek *et al.*, 1987).

At the same total concentration, the *in vitro* comparison of drug and metabolite mixtures at D1 and Css did not reveal a striking difference in their respective effects on MCF-7 and CAL-18 B. Thus reaching steady state, with its particular drug profile, would not be pharmacologically decisive for the anti-oestrogenic response. Moreover, in both $E_2R$ negative and positive cell lines, we noted that drug mixtures D1 and Css reduced cell proliferation as a function of drug concentration in the medium. Although it has not been demonstrated that a loading dose schedule would be better than the conventional TMX protocol (Abram, 1987), these pharmacological observations may comfort the thesis of authors calling for such a loading dose on the basis of pharmacokinetic arguments (Wilkinson *et al.*, 1982), so that efficient plasmatic levels would be reached quicker. On the other hand, the existence of this dose–response relationship in both $E_2R$ positive and negative cells may shed some light on the controversial literature data concerning the efficiency of high dose TMX (Manni & Arafah, 1981; Stewart *et al.*, 1982). Recently, Watkins (1988), increasing TMX dose to 90 mg daily in post-menopausal patients with advanced breast cancer progressing after standard dose, concluded that this high dose regimen was a clinically useful and highly acceptable approach. As a corollary, in one patient presenting an $E_2R$ positive recurrence during long-term TMX therapy, Jordan *et al.* (1987) noted serum levels of TMX and metabolites declining in the year prior to recurrence.

We examined serum levels of TMX and metabolites with regard to treatment response. The strict selection of patients led to a limited group of 13 patients. On this set, no significant correlation between treatment response and TMX and/or metabolite individual serum levels was found. Although 4-OHT appears to be the more potent drug tested *in vitro* on $E_2R$ positive cells, its plasma levels at steady state had never exceeded 69 nM and this patient did not respond to treatment. Moreover, in our total set of 78 patients, mean concentration of 4-OHT was $5\pm10$ nM. These levels of 4-OHT are very low to expect an antiproliferative effect, particularly on $E_2R$ negative tumours. Thus, from the present data it does not seem possible to draw conclusions on the importance of TMX metabolism in overall response *in vivo*. However, new anti-oestrogenic compounds have recently been developed on the basis of the 4-OHT structure, e.g. the promising 3-OH TMX (Loser *et al.*, 1985). Another potential approach would be to stimulate, by appropriate drug associations *in vivo*, the conversion of TMX in its more efficient hydroxylated form. This could be theoretically achievable when the enzymatic systems that process TMX and metabolites have been elucidated. The hitherto unknown biotransformation of metabolite Z in Y that we observed in the presence of tumour cells may also stimulate such future investigations.

### References

ABRAM, W.P. (1987). A multicentre trial evaluating loading doses of 'nolvadex' (Tamoxifen) in the treatment of advanced breast cancer. Proceedings of the 4th European Conference on Clinical Oncology and Cancer Nursing, Madrid, A447.

ADAM, H.K., PATTERSON, J.S. & KEMP, J.V. (1980). Studies of the metabolism and pharmacokinetics of tamoxifen in normal volunteers. *Cancer Treat. Rep.*, **64**, 761.

BAUM, M. & NATO. (1985). Controlled trial of tamoxifen as single agent in management of early breast cancer. *Lancet*, **i**, 836.

BRIAND, P. & LYKKESFELDT, A.E. (1984). Effect of estrogen and antiestrogen on the human breast cancer cell line MCF-7 adapted to growth at low serum concentration. *Cancer Res.*, **44**, 1114.

FISCHER, B., BROWN, A., WOLMARK, N. and 7 others (1987). Prolonging tamoxifen therapy for primary breast cancer. *Ann. Intern. Med.*, **106**, 649.

FURR, B.J.A. & JORDAN, V.C. (1984). The pharmacology and clinical uses of tamoxifen. *Pharmacol. Ther.*, **25**, 127.

GIOANNI, J., COURDI, A., LALANNE, C.M. and 5 others (1985). Establishment, characterization, chemosensitivity and radiosensitivity of two different cell lines derived from a human breast cancer biopsy. *Cancer Res.*, **45**, 1246.

GULINO, A., BARRERA, G., VACCA, A. and 5 others (1986). Calmodulin antagonism and growth-inhibiting activity of triphenylethylene antiestrogens in MCF-7 human breast cancer cells. *Cancer Res.*, **46**, 6274.

HAYWARD, J.L., RUBENS, R.D., CARBONE, P.P., HEUSON, C., KUMAOKA, S. & SEGALOFF, A. (1978). Assessment of response to therapy in advanced breast cancer. *Eur. J. Cancer*, **14**, 1291.

JORDAN, C.V. (1984). Biochemical pharmacology of antiestrogen action. *Pharmacol. Rev.*, **36**, 245.

JORDAN, C.V., BAIN, R.C., BROWN, R.B., GOSPEN, B. & SANTOS, M.A. (1983). Determination and pharmacology of a new hydroxylated metabolite of tamoxifen observed in patient sera during therapy for advanced breast cancer. *Cancer Res.*, **43**, 1446.

JORDAN, C.V., FRITZ, N.F. & TORMEY, D.C. (1987). Endocrine effects of adjuvant chemotherapy and long-term tamoxifen administration on node-positive patients with breast cancer. *Cancer Res.*, **47**, 624.

KATZENELLENBOGEN, B.S., MILLER, A.M., MULLICK, A. & SHEEN, Y.Y. (1985). Antiestrogen action in breast cancer cells: modulation of proliferation and protein synthesis, and interaction with estrogen receptors and additional antiestrogen binding sites. *Breast Cancer Res. Treat.*, **5**, 231.

KEMP, J.V., ADAM, H.K., WAKELING, A.E. & SLATER, R. (1983). Identification and biological activity of tamoxifen metabolites in human serum. *Biochem. Pharmacol.*, **32**, 2045.

KNABBE, C., LIPPMAN, M.E., WAKEFIELD, L.M. and 4 others (1987). Evidence that transforming growth factor $\beta$ is a hormonally regulated negative growth factor in human breast cancer cells. *Cell*, **48**, 417.

LIEN, E.A., SOLHEIM, E., KVINNSLAND, S. & VELAND, P.M. (1988). Identification of 4-hydroxy-N-desmethyltamoxifen as a metabolite of Tamoxifen in human bile. *Cancer Res.*, **48**, 2304.

LOSER, R., SIEBEL, K., KOO, W. & EPPENBURGER, V. (1985). *In vivo* and *in vitro* antiestrogenic action of 3-hydroxytamoxifen, tamoxifen and 4-hydroxytamoxifen. *Eur. J. Cancer Clin. Oncol.*, **21**, 985.

MANNI, A. & ARAFAH, B.M. (1981). Tamoxifen-induced remission in breast cancer by escalating the dose to 40 mg daily after progression on 20 mg daily: a case report and review of the literature. *Cancer*, **48**, 873.

MILANO, G., ETIENNE, M.C., FRENAY, M. and 7 others (1987). Optimised analysis of tamoxifen and its main metabolites in the plasma and cytosol of mammary tumours. *Br. J. Cancer*, **55**, 509.

MILANO, G., MOLL, J.L., FORMENTO, J.L. and 5 others (1983). Simultaneous micromeasurement of steroid receptors in breast cancer. *Br. J. Cancer*, **48**, 579.

MILLER, M.A., LIPPMAN, M.E. & KATZENELLENBOGEN, B.S. (1984). Antiestrogen binding in antiestrogen growth-resistant estrogen-responsive clonal variants of MCF-7 human breast cancer cells. *Cancer Res.*, **44**, 5038.

REDDEL, R.R., MURPHY, L.C., HALL, R.E. & SUTHERLAND, R.L. (1985). Differential sensitivity of human breast cancer cell lines to the growth-inhibitory effects of tamoxifen. *Cancer Res.*, **45**, 1525.

REDDEL, R.R., MURPHY, L.C. & SUTHERLAND, R.L. (1983). Effects of biologically active metabolites of tamoxifen on the proliferation kinetics of MCF-7 human breast cancer cells *in vitro*. *Cancer Res.*, **43**, 4618.

ROCHEFORT, H. (1987). Do antiestrogens and antiprogestins act as hormone antagonists or receptor-targeted drugs in breast cancer? *Trends Pharmacol. Sci.*, **8**, 126.

SHEK, L.L.M., GODOLPHIN, W. & SPINELLI, J.J. (1987). Oestrogen receptors, nodes and stage as predictors of post-recurrence survival in 457 breast cancer patients. *Br. J. Cancer*, **56**, 825.

STEWART, J.F., MINTON, M.J. & RUBENS, R.D. (1982). Trial of tamoxifen at a dose of 40 mg daily after disease progression during tamoxifen therapy at a dose of 20 mg daily. *Cancer Treat. Rep.*, **66**, 1445.

SU, H.D., MAZZEI, G.J., VOGLER, W.R. & KUO, J.F. (1985). Effect of tamoxifen, a non-steroidal antiestrogen, on phospholipid/calcium-dependent protein kinase and phosphorylation of its endogenous substrate proteins from the rat brain and ovary. *Biochem. Pharmacol.*, **34**, 3649.

TAYLOR, C.M., BLANCHARD, B. & ZAVA, D.T. (1984). Estrogen receptor-mediated and cytotoxic effects of the antiestrogens tamoxifen and 4-hydroxytamoxifen. *Cancer Res.*, **44**, 1409.

VOGEL, C.L., EAST, D., VOIGT, W. & THOMSEN, S. (1987). Response to tamoxifen in estrogen receptor-poor metastatic breast cancer. *Cancer*, **6**, 1184.

WAKELING, A.E. & SLATER, S.R. (1980). Estrogen-receptor binding and biological activity of tamoxifen and its metabolites. *Cancer Treat. Rep.*, **64**, 741.

WATKINS, S.M. (1988). The value of high dose tamoxifen in postmenopausal breast cancer patients progressing on standard doses: a pilot study. *Br. J. Cancer*, **57**, 320.

WILKINSON, P.M., RIBEIRO, G.G., ADAM, H.K., KEMP, J.V. & PATTERSON, J.S. (1982). Tamoxifen (Novaldex) therapy-rationale for loading dose followed by maintenance dose for patients with metastatic breast cancer. *Cancer Chemother. Pharmacol.*, **10**, 33.